# Comparing the diagnostic value of 18F-FDG PET/CT scan and bone marrow biopsy in newly diagnosed pediatric neuroblastoma and ganglioneuroblastoma

**DOI:** 10.3389/fonc.2022.1031078

**Published:** 2022-12-15

**Authors:** Zheng Fu, Jiazhong Ren, Jing Zhou, Junkang Shen

**Affiliations:** ^1^ Department of Radiology, The Second Affiliated Hospital of Soochow University, Suzhou, China; ^2^ Department of Imaging Center, Shandong Cancer Hospital and Institute, Shandong First Medical University, Shandong, China; ^3^ Department of Radiotherapy, Shandong Cancer Hospital and Institute, Shandong First Medical University, Shandong, China

**Keywords:** NB, GNB, ^18^F-FDG PET/CT, bone marrow biopsy, bone–bone marrow involvement

## Abstract

**Objective:**

This study aims to compare the diagnostic value of ^18^F-fluorodeoxyglucose (18-FDG) positron emission tomography (PET)/computed tomography (CT) (^18^F-FDG PET/CT) scan and bone marrow biopsy (BMB) for evaluating bone marrow infiltration (BMI) in newly diagnosed pediatric neuroblastoma (NB) and ganglioneuroblastoma (GNB).

**Methods:**

We retrospectively reviewed 51 patients with newly diagnosed NB and GNB between June 1, 2019 and May 31, 2022. Each patient had undergone ^18^F-FDG PET/CT and BMB within 1 week and received no treatment. Clinical data were collected and statistically analyzed, including age, sex, pathologic type, and laboratory parameters. ^18^F-FDG PET/CT and BMB revealed the result of bone lesions.

**Results:**

A concordance analysis showed that, in this study population, ^18^F-FDG PET/CT and BMB were in moderate agreement (Cohen’s Kappa = 0.444; *p* = 0.001), with an absolute agreement consistency of 72.5% (37 of 51). The analysis of the receiver operating characteristic (ROC) curve determined that the areas under the ROC curve (AUCs) of SUV_BM_ and SUV/HE-SUVmax were 0.971 (95% CI: 0.911–1.000; *p* < 0.001) and 0.917 (95% CI: 0.715–1.000; *p* < 0.001) to predict bone–bone marrow involvement (BMI), respectively.

**Conclusion:**

^18^F-FDG PET/CT detects BMI with good diagnostic accuracy and can reduce unnecessary invasive inspections in newly diagnosed pediatric NB and GNB, especially patterns C and D. The analysis of the semi-quantitative uptake of ^18^F-FDG, including SUV_BM_ and SUV_BM_/HE-SUVmax, enables an effective differentiation between patterns A and B.

## 1 Introduction

Neuroblastoma is the most common pediatric solid tumor occurring in the sympathetic nervous system and accounts for approximately 15% of childhood cancer‐related mortality (1). A total of 70% of patients with NB have metastatic disease at the time of diagnosis, which commonly involves the cortical bone and the bone marrow (BM). However, metastatic bone–BM is a sign of advanced disease and implies a poor prognosis (2). Neuroblastoma presents a great heterogeneity in clinical behavior and survival rates; therefore, accurate staging is crucial to choose the appropriate treatment.

Bone marrow biopsy (BMB) is currently a “gold standard” modality in identifying bone marrow involvement (BMI) due to its advantages in diagnosis, staging, and treatment monitoring in childhood malignancies (3). BMB is obtained from the dorsal portion of the iliac crest and is the most easily accessible approach for BM evaluation. It is based on the assumption that, in cases of BMI, tumor cells spread non-focally through the bone–BM (4), which was demonstrated to be incorrect. However, it is a painful and invasive procedure, especially for children. More worrying still is the fact that a part of the patients repeatedly required BM punctures during the treatment. Additionally, the major drawback of BMB is that it may miss focal NB tumor cell deposits and bone metastases in areas far from the iliac bone because it yields information from a limited area (3). Using BMB to determine metastatic bone–BM is insufficient. Thus, another examination is needed to supplement the deficiency that BMB presents.

Nowadays, iodine-123 metaiodobenzylguanidine (^123^I-MIBG) scintigraphy is a mainstay method in pediatric NB (5). Nevertheless, ^123^I-MIBG scintigraphy imaging has several disadvantages such as no concentration of MIBG in 10% of tumors, limited spatial resolution, and limited sensitivity in small lesions. ^18^F-fluorodeoxyglucose (^18^F-FDG) positron emission tomography (PET)/computed tomography (CT) (^18^F-FDG PET/CT) is commonly used to complete the staging and prognosis prediction of malignant tumors and can also be used to evaluate marrow infiltration. Compared with ^123^I-MIBG scintigraphy imaging, the superiorities of PET are high ^18^F-FDG avidity of the BM and better identification of FDG abnormalities in the BM and bone (6, 7). Moreover, an important advantage of PET/CT over BMB is that PET/CT can assess all BM sites at once and find unintended BMI in areas where biopsies are not usually performed (8), thus avoiding the sampling limitation of BMB. ^18^F-FDG PET/CT has a good overall diagnostic accuracy with high sensitivity and specificity in the detection of bone or BMI in pediatric neuroblastoma (9, 10). The sample size of the study was small, and further research is needed (10).

Many investigators are concerned that BM FDG uptake may mask or mimic metastatic disease due to the initial use of chemotherapy or granulocyte colony-stimulating factor in NB patients, thereby reducing the sensitivity of ^18^F-FDG PET/CT scan (11, 12). To reduce this adverse effect, we compared ^18^F-FDG PET/CT and BMB in the assessment of BMI in patients with NB before receiving chemotherapy. Therefore, our retrospective analysis aimed to explore the diagnostic performance of ^18^F-FDG PET/CT in detecting BMI in newly diagnosed neuroblastoma (NB) and ganglioneuroblastoma (GNB).

## 2 Methods

### 2.1 Patients

We retrospectively collected all the data of patients with neuroblastoma (age <18 years) who had undergone ^18^F-FDG PET/T before treatment from June 1, 2019, to May 31, 2022. There were 51 patients included, including 41 patients with NB and 10 with GNB. They were diagnosed for the first time and underwent both ^18^F-FDG PET/CT and BMB within 1 week. Patients who had undergone any treatment procedures before ^18^F-FDG PET/CT were excluded.

The data obtained from the clinical medical records included age, sex, pathologic type, and laboratory parameters, such as lactate dehydrogenase (LDH), neuron-specific enolase (NSE), serum albumin (A), serum total protein (TP), hemoglobin (Hb), and blood platelet (BP). All methods were performed according to the relevant guidelines and regulations.

Pediatric neuroblastoma usually has three histological types: NB, GNB, and ganglioneuroma (GN). We excluded GN because it is a benign tumor (13) and does not present with bone metastasis.

### 2.2 ^18^F-FDG PET-CT imaging protocol

^18^F-FDG is produced on a MiniTrace Cyclotron and automatic synthesis system of GE Healthcare, with a radiochemical purity of more than 95%. The patients fasted for at least 6 h before the examination and had blood glucose lower than 10 mmol/L. The intravenous injection of FDG ranged from 4.44 to 5.55 MBq/kg. Thirty-six patients were given oral sedation for PET scans. PET/CT scans were performed 60 minutes after injecting radiolabeled ^18^F-FDG using a Siemens PET/CT system (Horizon). The examinations included a head-to-toe CT scan (80 kV; 50–100 mAs) and a three-dimensional (3D) PET scan (2 min per bed; six to seven beds). The rotation time was 0.6. The slice thickness was 3.75 mm. The increment was 3.27. The pitch was 0.984. The images were displayed on the Syngo.via workstation.

### 2.3 ^18^F-FDG PET/CT image analysis

#### 2.3.1 Patterns of ^18^F-FDG uptake in bone marrow

All scans were read independently by two experienced nuclear medicine physicians who were blinded to clinical information, laboratory assessments, and BMB results. A third reader adjudicated the discrepancies.

**Figure 1:**
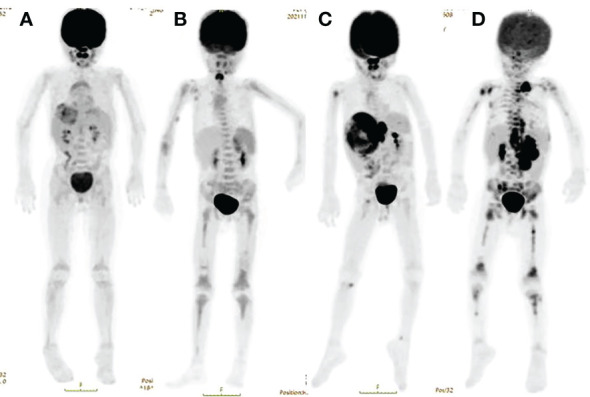
Different patterns of 18F-FDG PET-CT bone-bone marrow uptake of 18F-FDG.

The BM uptake was categorized (14, 15) (**Figure 1**). “A” was defined as no increased ^18^F**-**FDG uptake, “B” was defined as diffusely increased ^18^F**-**FDG uptake, “C” was defined as focal/multifocal only, which was one or more circumscribed areas of increased ^18^F**-**FDG uptake within the skeleton, “D” was defined as combined focal/multifocal, and diffuse ^18^F**-**FDG uptake appeared. Cases where BMI lesions appeared to be caused by a contiguous spread from the adjacent soft tissues were considered negative. The presence of osseous sclerosis at sites of uptake on the CT component indicating bone metastases was also recorded. However, the diffuse BM ^18^F**-**FDG uptake with patterns A and B was less likely to be diagnosed as BMI because this may also result from paraneoplastic bone marrow activation (16).

#### 2.3.2 Semi-quantitative ^18^F-FDG uptake

The maximum standardized uptake value (SUVmax) of the most hypermetabolic bone–BMI was measured (BM-SUVmax) in patterns C and D. Additionally, a circular region of interest (ROI) was drawn inside the normal area in segments VII and VIII of the liver. The average hepatic SUVmax was recorded (HE-SUVmax). To decrease the potential age-related differences, the ratio of BM-SUVmax to HE-SUVmax (BM-SUVmax:HE-SUVmax) was calculated. Patients with hepatic metastases and those with underlying destructive osseous lesions shown on the CT were excluded.

To assess the role of semi-quantitative ^18^F-FDG uptake in BM in differentiating BMI from normal or reactive BM, the SUVmax of BM (SUV_BM_) was measured in patients with normal or homogeneously increased FDG uptake in BM (patterns A and B). A spherical volume of interest corresponding to the variable vertebral sizes of subjects was placed over the central part of the L5 vertebral body, and SUV_BM_ was measured. If L5 vertebrae were abnormal, in cases of tumor direct involvement or fracture, an alternative measurement of the L4 vertebral body was obtained, and so on. To account for the potential age-related differences, SUV_BM_ was further normalized by hepatic uptake (SUV_BM_/HE-SUVmax) (14, 15).

### 2.4 Marrow disease identification using iliac crest trephine biopsy

A pediatric oncologist at our hospital took BMB from only the standard regions of the posterior superior iliac and no other sites. The biopsy results were reported based on histopathology, and immunophenotyping was evaluated by the oncology and pathology departments.

### 2.5 Identification of bone marrow involvement

Patients with BM uptake (patterns C and D) were considered BMI-positive on the ^18^F-FDG PET/CT scan (16). A final diagnosis of BMI was determined if BMB was positive if the focal/multifocal intense FDG uptake in BM on PET was confirmed by directed biopsy or supplementary targeted magnetic resonance imaging (MRI) or the resolution of the BM lesions in parallel with other lesions on the follow-up ^18^F-FDG PET/CT scan. The typical focal/multifocal pattern is easier to diagnose in children, as degenerative diseases are less likely to occur in children but are rather common in adult patients (16). For future analysis, patients were divided into four groups: PET-positive BMB-positive, PET-positive BMB-negative, BMB-positive PET-negative, and PET-negative BMB-negative. Ultimately, according to the results, the patients were divided into the bone–BMI group and the non-bone–BMI group.

### 2.6 Statistical analysis

Statistical analyses were performed using SPSS software (version 28.0 for Windows; SPSS Inc.). Continuous data were described as the mean ± standard deviation (mean ± SD) or median and interquartile, depending on whether they followed a normal distribution. The categorical variables were described as numbers. The degree of agreement between BMB and ^18^F-FDG PET/CT was assessed using Cohen’s Kappa. A *k*-value of 0.0–0.39 represented a slight agreement, 0.4–0.74 was moderate, and 0.75–1.0 was almost perfect. Differences between groups were compared using the Mann–Whitney *U*-tests for the continuous variables and chi-square tests and Fisher’s exact test for the categorical variables. Receiver operating characteristic (ROC) curves were used to determine the cutoff values of BM-SUVmax to predict BMI in patients with normal or homogenously increased BM ^18^F-FDG uptake. All tests were two-sided, and a probability of less than 0.05 was considered statistically significant.

## 3 Results

### 3.1 Patients’ characteristics

We retrospectively studied 51 patients with NB and GNB (age <18 years). This study included 22 girls and 29 boys, with an average age of 37.18 months. The general characteristics of the patients are summarized in **Table 1**. Among the 41 patients with NB, 27 (65.9%) had a final diagnosis that showed BMI. Among the 10 patients with GNB, eight (80%) had a final diagnosis that showed BMI. The status of bone–BMI in patients with NB and GNB (*χ* ^2^ = 0.747; *p* = 0.387) is shown in **Table 2**.

**Table 1:** General characteristics of 51 patients.

Characteristics		Number of patients	%
Age (months)	Median (range)	36 (2–144)	
Gender	Male	29	43.1
	Female	22	56.9
Histology	Neuroblastoma	41	80.4
	Ganglioneuroblastoma	10	19.6
Location	Neck	1	2.0
	Chest	5	9.8
	Abdomen	44	86.3
	Pelvis	1	2.0
	Negative	15	62.5
BMB	Positive	31	60.8
	Negative	20	39.2
PET (BMI)^a^	Positive	27	52.9
	Negative	24	47.1
Total bone/bone–bone marrow involvement (BMI)^b^	Positive	36	70.6
	Negative	15	29.4

^a18^F-FDG PET/CT scan results.

^b^Eventually bone–BMI results.

**Table 2:** Status of bone–BMI in patients with neuroblastoma (NB) and ganglioneuroblastoma (GNB).

	NB	GNB	Total
Bone/BMI-positive	27 (52.9%)	8 (15.7%)	35 (68.6%)
Bone/BMI-negative	14 (27.5%)	2 (3.9%)	16 (31.4%)
Total	41 (80.4%)	10 (19.6%)	51 (100%)

### 3.2 The results of the bone assessment using ^18^F-FDG PET/CT and bone marrow biopsy

A final diagnosis of BMI was confirmed using directed biopsy or other multimodality imaging results, especially those of BM uptake patterns A and B.

The mean *κ* coefficient was 0.444 for BMB and ^18^F-FDG PET/CT agreement for BM uptake, indicating a moderate level of agreement (*p* = 0.001) with an absolute agreement consistency of 72.5% (37 of 51). **Table 3** summarizes the bone–BM assessment results of ^18^F-FDG PET/CT and BMB. The distribution of the uptake ^18^F-FDG pattern in the bone–BM in different pathological subtypes and the results of BMB are shown in **Table 4**.

**Table 3:** Bone–BM assessment results of ^18^F-FDG PET/CT and bone marrow biopsy (BMB).

	PET-positive	PET-negative	Total
BMB-positive	22 (43.1%)	9 (17.6%)	31 (60.8%)
BMB-negative	5 (9.8%)	15 (29.4%)	20 (39.2%)
Total	27 (52.9%)	24 (47.1%)	51 (100%)

**Table 4:** Distribution of the absorbed 18F-FDG pattern in the bone–bone marrow in the different pathological subtypes and bone marrow biopsy (BMB) results.

Pattern	Pathology		Total	BMB result		Total
	NB	GNB		BMB-positive	BMB-negative
A	20 (39.2%)	4 (7.8%)	24 (47.1%)	9 (17.6%)^a^	15 (29.4%)^a^	24 (47.0%)
B	5 (9.8%)	1 (2.0%)	6 (11.8%)	5 (9.8%)	1 (2.0%)	6 (11.8%)
C	7 (13.7%)	0 (0%)	7 (13.7%)	3 (5.9%)	4 (7.8%)	7 (13.7%)
D	9 (17.6%)	5 (9.8%)	14 (27.5%)	14 (27.5%)	0 (0%)	14 (27.5%)
Total	41 (80.4%)	10 (19.6%)	51 (100%)	31 (60.8%)	20 (39.2%)	51 (100%)

a PET-positive.

### 3.3 Semi-quantitative analysis of bone marrow ^18^F-FDG uptake pattern

**Figure 2:**
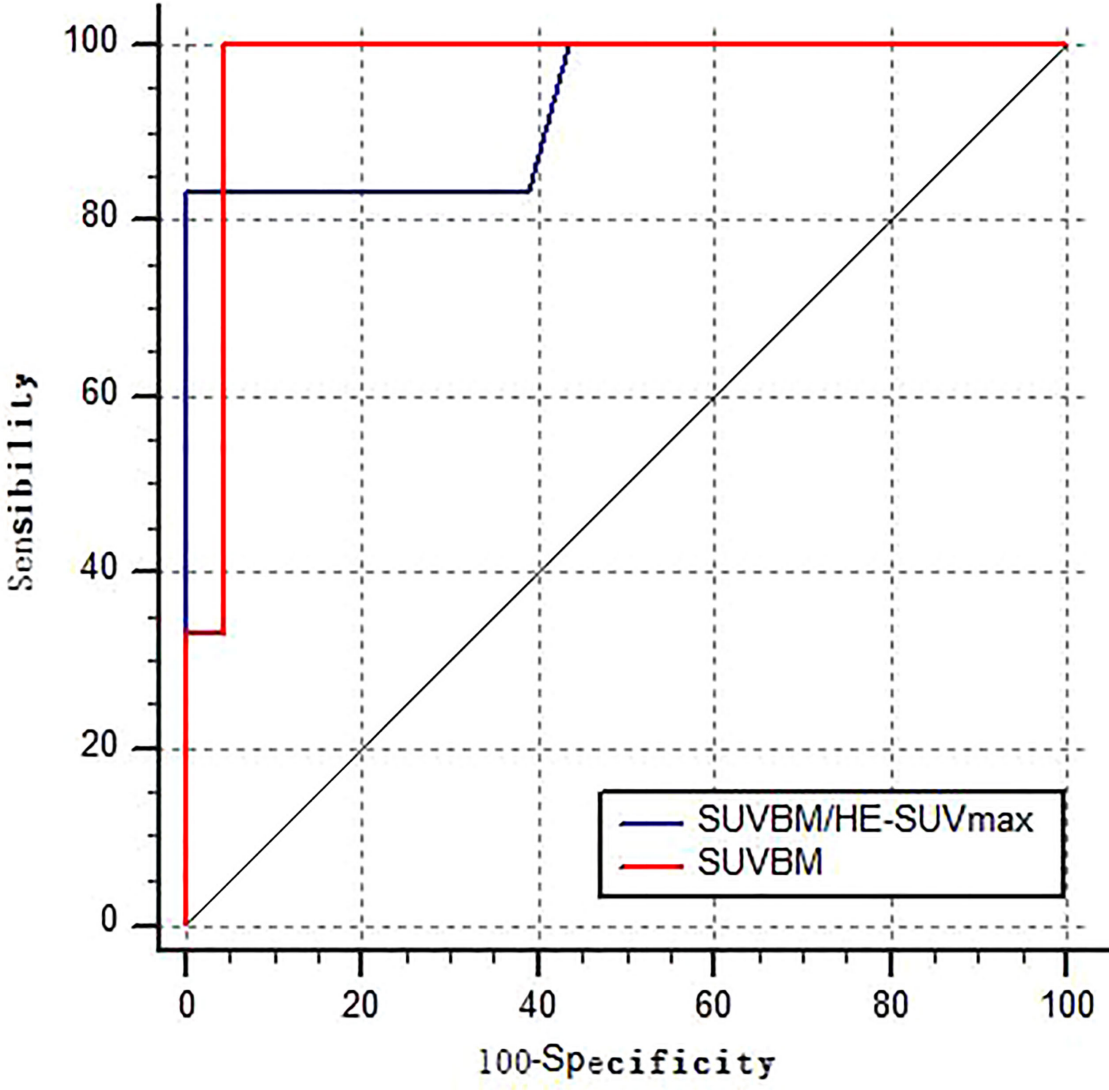
Receiver operating characteristic curve of SUVBM and SUVBM/HE-SUVmax performance in the detection of BMI in NB and GNB patients in patterns A–B.

BM semi-quantitative ^18^F-FDG uptake was evaluated in 30 patients with patterns A and B. Eventually, 24 patients had pattern A, and six had pattern B. Their SUV_BM_/HE-SUVmax showed no statistically significant difference (*Z* = -0.62; *p* > 0.05), and SUV_BM_ showed a statistically significant difference (*Z* = -3.5; *p* < 0.01). The ROC curve analyses determined that the AUCs of SUV_BM_ and SUV/HE-SUVmax were 0.971 (95% CI: 0.911–1.000; *p* < 0.001) and 0.917 (95% CI: 0.715–1.000; *p* < 0.001), respectively, for predicting bone–BMI (**Figure 2**). According to the Youden index, a cutoff value of 1.89 for SUV_BM_ was determined as the point with a maximum sum of sensitivity (100%) and specificity (95.65%). A cutoff value of 1.66 for SUV_BM_/HE-SUVmax was determined as the point with a maximum sum of sensitivity (80%) and specificity (100%). In patterns C and D, the AUCs of BM-SUVmax and BM-SUVmax/HE-SUVmax were 0.398 (95% CI: 0.156–0.640; *p* = 0.456) and 0.582 (95% CI: 0.319–0.844; *p* = 0.551), respectively, for predicting bone–BMI.

### 3.4 Clinical feature variables and status of bone marrow involvement

The different clinical features were compared among patients with different BM ^18^F-FDG uptake patterns. Patients with patterns of A and B showed statistically significant differences in terms of NSE and Hb. In the semi-quantitative analysis of ^18^F-FDG PET/CT, SUV_BM_ and SUV/HE-SUVmax also showed statistically significant differences. The different clinical features in patients with patterns C and D showed no statistically significant differences. The various clinical features and the semi-quantitative analysis of ^18^F-FDG PET/CT in patients with different BM ^18^F-FDG uptake patterns are shown in **Table 5**.

**Table 5:** Various clinical features and semi-quantitative ^18^F-FDG PET/CT in patients with different patterns of bone–bone marrow.

Characteristics	^18^F-FDG uptake patterns of BM					
	A	B	*P*	C	D	*P*
Age(months)	22 (2–67)	45 (31–84)	**<0.05** ^a^	51 (18–108)	37 (11–144)	>0.05
Gender (M:F)	13:11	3:3	>0.05	5:2	8:6	>0.05
LDH, IU/L	488 (354, 1,638)	613.5 (543.5, 1,826.5)	>0.05	1,251 (507, 3,273)	689.5 (567.25, 1,381.75)	>0.05
NSE, ng/L	133 (53.6, 498)	237.19 ± 450.93	**<0.05** ^a^	419.96 ± 236.37	394 (231, 600)	>0.05
A, g/L	45.67 ± 5.20	42.72 ± 10.21	>0.05	40.04 ± 1.74	37.20 ± 3.74	>0.05
TP, g/L	69.06 ± 7.07	71.80 ± 9.93	>0.05	39.7 (38.4, 41.6)	66.08 ± 4.60	>0.05
Hb, g/L	103.39 ± 18.11	81.67 ± 8.76	**<0.05** ^a^	96.29 ± 20.28	83.07 ± 9.16	>0.05
BP,10^9^/L	366.52 ± 188.72	297.00 ± 163.35	>0.05	313.57 ± 152.09	258.50 ± 118.98	>0.05
HE-SUVmax	1.33 (1.03, 1.60)	1.41 ± 0.36	>0.05	1.39 ± 0.56	1.82 ± 0.83	>0.05
SUV_BM_	1.27 ± 0.41	1.24 (1.0, 1.56)	**<0.05** ^a^			
SUV_BM_/HE-SUVmax	0.99 ± 0.30	2.06 ± 0.79	**<0.05** ^a^			
BM-SUVmax				4.01 ± 0.76	4.85 ± 1.82	>0.05
BM-SUVmax/HE-SUVmax				3.21 ± 1.07	3.0 ± 1.33	>0.05

a The meaning of the bold values wanted to emphasize p < 0.05, the difference is statistically significant in P <0.05.

## 4 Discussion

Many pediatric malignancies usually have bone–BMI, such as lymphoma and neuroblastoma (17, 18). In neuroblastoma, accurate staging is essential for prognosis and choosing appropriate treatment options (19). ^18^F-FDG PET/CT has become an available routine clinical practice, greatly modifying the diagnosis and staging approach of neuroblastoma. Neuroblastoma is often characterized by a high ^18^F-FDG uptake (19), including bone–BMI. Compared with traditional CT or MRI examination, ^18^F-FDG PET/CT could provide important information about neuroblastoma and show more extensive disease in one examination.

Research revealed that ^18^F-FDG PET/CT was more sensitive than BMB for detecting BMI in pediatric lymphoma staging and can even replace BMB (15, 17, 20). However, the current protocol calls for screening for BMI using BMB as the standard method for evaluating pediatric neuroblastoma. BMB is an invasive and expensive procedure, which samples only a limited part of the BM (17). Evaluating BMI using ^18^F-FDG PET/CT would unduly reduce the risk of staging (8). In their meta-analysis of seven studies, Sun et al. reported that ^18^F-FDG PET/CT has excellent overall diagnostic accuracy and high sensitivity and specificity in detecting bone–BMI in pediatric neuroblastoma (10). However, no study compared the diagnostic value of ^18^F-FDG PET/CT and BMB in detecting metastatic bone–BM in newly diagnosed pediatric neuroblastoma.

In our study, we used both ^18^F-FDG PET/CT and BMB, and 22 patients (43.1%; 22 of 51) were consistently considered BMI-positive, and 15 patients (29.4%; 15 of 51) were BMI-negative. However, 14 patients showed inconsistent results. After undergoing BMB and ^18^F-FDG PET/CT, nine were BMB-positive and ^18^F-FDG PET/CT-negative (seven NB and two GNB patients). In contrast, the remaining five patients with inconsistent results were BMB-negative and ^18^F-FDG PET/CT-positive (all NB patients). The concordance analysis showed a moderate agreement between ^18^F-FDG PET/CT and BMB in this study population (Cohen’s *k* = 0.444; *p* = 0.001), with an absolute agreement consistency of 72.5% (37 of 51). We found that ^18^F-FDG PET/CT and BMB are complementary in the evaluation of BMI in patients with neuroblastoma.

^18^F-FDG PET-CT represents 100% positivity in bone–BMI, which allows avoiding BMB in patients with pattern D. In some patients with patterns B and C, ^18^F-FDG PET/CT showed an abnormal focal bone–BMI at the acetabular bone or disease at other sites (*e*.*g*., femur, humerus). This phenomenon may be due to the BMB examining the iliac bone lesion, which provides information from a limited area. Thus, BMB is not perfect as a method for evaluating BMI in patients with patterns B and C. However, in patients with pattern C, ^18^F-FDG PET/CT can well identify bone involvement. There may be new value in improving bone–BMI uptake patterns for further research.

Meanwhile, a correct diagnosis of diffuse BM ^18^F-FDG uptake was a challenge in pediatric lymphoma (14, 15). Under normal conditions, the BM shows a homogenous low uptake of ^18^F-FDG, less intense than the liver. A diffuse BM ^18^F-FDG uptake can occur because of different reasons: malignancies or hematopoietic diseases, an inflammatory response, paraneoplastic BM activation as a result of recent chemotherapy, or administration of hematopoietic growth factors (21). In our study, 24 patients with pattern A showed no ^18^F-FDG uptake, and six patients with pattern B showed diffuse ^18^F-FDG uptake. Nine of 24 (20.8%) patients with pattern A and five of six (83.3%) patients with pattern B were BMB-positive.

In our study, to further distinguish between patterns A and B and assess the value of semi-quantitative ^18^F-FDG uptake, the L5 vertebrae SUV _BM_ was measured in patients with normal or homogeneously increased ^18^F-FDG uptake in BM. In patients with pattern A, only 11 had an L5 vertebrae SUV_BM_ lower than that of the liver (45.8%), but 13 patients showed a SUV_BM_ lower than 1.5 times that of the liver (54.2%). In patients with pattern B, all had an L5 vertebrae SUV_BM_ greater than that of the liver (100%). In contrast to model A, five model B patients had greater than 1.5 times as many livers (83.3%). From the ROC curve analyses, we found that the AUCs of SUV_BM_ and SUV_BM_/HE-SUVmax were 0.971 (95% CI: 0.911–1.000; *p* < 0.001) and 0.917 (95% CI: 0.715–1.000; *p* < 0.001), respectively, for predicting BMI. These ^18^F-FDG PET/CT metabolic parameters can effectively differentiate between patterns A and B. Our data showed that the L5 vertebrae SUV_BM_ range was 0.47–2.1 in patients aged 2–67 months, consistent with the normal range of 1.3–2.1 in patients aged 2–15 months (22). Thus, the analysis of the semi-quantitative uptake of ^18^F-FDG, including SUV_BM_ and SUV/HE-SUVmax, allows an effective differentiation between patterns A and B.

In patterns of C and D, ^18^F-FDG PET/CT was more accurate for the diagnosis of bone–BMI. In pattern D, the diagnostic agreement rate for ^18^F-FDG PET/CT and BMB was 100%. In pattern C, the diagnostic inconsistency rate was 71.4%, and BMB had a high false negative. The ROC curve analyses showed that the AUCs of BM-SUVmax and BM-SUVmax/HE-SUVmax were 0.398 (95% CI: 0.156–0.640; *p* = 0.456) and 0.582 (95% CI: 0.319–0.844; *p* = 0.551), respectively, for predicting BMI. The ROC curve analyses cannot distinguish between patterns C and D. ^18^F-FDG PET/CT-guided BMB should be performed to reduce the harm of unnecessary BM aspiration biopsies in patients with pattern C (23).

Clinical features including age, NSE, and Hb showed statistically significant differences between patterns A and B. Many patients with pattern A have normal Hb, while patients with pattern B have low hemoglobin. Moreover, no statistically significant clinical indicators were found for patients with patterns C and D. Thus, clinical features offer greater advantages in diagnosing patterns A and B.

The main limitation of this study is its retrospective nature because we were unable to biopsy the bone lesions or perform other imaging examinations of bone lesions with ^18^F-FDG uptake. There is also the possibility of false negatives in some post-chemotherapy follow-up patients, particularly for pattern A.

## 5 Conclusion

This study suggests that ^18^F-FDG PET/CT can detect BMI with good diagnostic accuracy and reduce unnecessary invasive inspections in newly diagnosed pediatric NB and GNB, especially for patients with patterns C and D. For patients with pattern D, ^18^F-FDG PET/CT can replace BMB.

## Data availability statement

The original contributions presented in the study are included in the article/supplementary material. Further inquiries can be directed to the corresponding authors.

## Ethics statement

The study was conducted from a new perspective and with a new approach. Written informed consent to participate in this study was provided by the participants’ legal guardian/next of kin. Written informed consent was obtained from the minor(s)’ legal guardian/next of kin for the publication of any potentially identifiable images or data included in this article.

## Author contributions

ZF and JS conceived and designed the study and helped to draft the manuscript. JZ performed the data collection. JR performed the statistical analysis. All authors contributed to the article and approved the submitted version.

## References

[1] Tsubota S Kadomatsu K. Origin and initiation mechanisms of neuroblastoma. Cell Tissue Res (2018) 372:211–21. doi: 10.1007/s00441-018-2796-z 29445860

[2] Cohn SL Pearson AD London WB Monclair T Ambros PF Brodeur GM. The international neuroblastoma risk group (INRG) classification system: an INRG task force report. J Clin Oncol (2009) 27:289–97. doi: 10.1200/JCO.2008.16.6785 PMC2650388 19047291

[3] Yağci KB Koçyiğit DE Adamhasan F Küpeli S. The value of 18F-FDG PET/CT in detecting bone marrow involvement in childhood cancers. J Pediatr Hematol Oncol (2019) 41(6):438–41. doi: 10.1097/MPH.0000000000001499 31033787

[4] Orr KE McHugh K. The new international neuroblastoma response criteria. Pediatr Radiol (2019) 49:1433–40. doi: 10.1007/s00247-019-04397-2 31620844

[5] Liu CJ Lu MY Liu YL Ko CL Ko KY Tzen KY. Risk stratification of pediatric patients with neuroblastoma using volumetric parameters of 18F-FDG and 18F-DOPA PET/CT. Clin Nucl Med (2017) 42:e142–e8. doi: 10.1097/RLU.0000000000001529 28072621

[6] Brian HK Henry WY Stevn M L Kim K Nai KC. Extending positron emission tomography scan utility to high-risk neuroblastoma: fluorine-18 fluorodeoxyglucose positron emission tomography as sole imaging modality in follow-up of patients. J Clin Oncol (2001) 19(14):3397–405. doi: 10.1200/JCO.2001.19.14.3397 11454888

[7] Wang Y Xu Y Kan Y Wang W Yang J. Diagnostic value of seven different imaging modalities for patients with neuroblastic tumors: A network meta-analysis. Contrast Media Mol Imaging (2021) 2021:5333366. doi: 10.1155/2021/5333366 34548851 PMC8429030

[8] Adams HJ Kwee TC de Keizer B Fijnheer R de Klerk JM Littooij AS. Systematic review and meta-analysis on the diagnostic performance of FDG-PET/CT in detecting bone marrow involvement in newly diagnosed Hodgkin lymphoma: is bone marrow biopsy still necessary? Ann Oncol (2014) 25:921–7. doi: 10.1093/annonc/mdt533 24351400

[9] Ishiguchi H Ito S Kato K Sakurai Y Kawai H Fujita N. Diagnostic performance of (18)F-FDG PET/CT and whole-body diffusion-weighted imaging with background body suppression (DWIBS) in detection of lymph node and bone metastases from pediatric neuroblastoma. Ann Nucl Med (2018) 32:348–62. doi: 10.1007/s12149-018-1254-z PMC5970256 29667143

[10] Sun L Zhang B Peng R. Diagnostic performance of (18)F-FDG PET(CT) in bone-bone marrow involvement in pediatric neuroblastoma: A systemic review and meta-analysis. Contrast Media Mol Imaging (2021) 2021:8125373. doi: 10.1155/2021/8125373 34220381 PMC8221854

[11] Taggart DR Han MM Quach A Groshen S Ye W Villablanca JG. Comparison of iodine-123 metaiodobenzylguanidine (MIBG) scan and [18F]fluorodeoxyglucose positron emission tomography to evaluate response after iodine-131 MIBG therapy for relapsed neuroblastoma. J Clin Oncol (2009) 27:5343–9. doi: 10.1200/JCO.2008.20.5732 PMC2773221 19805691

[12] Sharp SE Shulkin BL Gelfand MJ Salisbury S Furman WL. 123I-MIBG scintigraphy and 18F-FDG PET in neuroblastoma. J Nucl Med (2009) 50:1237–43. doi: 10.2967/jnumed.108.060467 19617326

[13] Wen GH Yu Y Wen T Rong C Gang R. Clinical and biological features of neuroblastic tumors: A comparison of neuroblastoma and ganglioneuroblastoma. Oncotarget (2017) 8(23):37730–9. doi: 10.18632/oncotarget.17146 PMC5514944 28465480

[14] Chen S Wang S He K Ma C Fu H Wang H. PET/CT predicts bone marrow involvement in paediatric non-Hodgkin lymphoma and may preclude the need for bone marrow biopsy in selected patients. Eur Radiol (2018) 28:2942–50. doi: 10.1007/s00330-018-5306-5 29383519

[15] Hassan A Siddique M Bashir H Riaz S Wali R Mahreen A. (18)F-FDG PET-CT imaging versus bone marrow biopsy in pediatric hodgkin's lymphoma: a quantitative assessment of marrow uptake and novel insights into clinical implications of marrow involvement. Eur J Nucl Med Mol Imaging (2017) 44:1198–206. doi: 10.1007/s00259-017-3647-y 28229191

[16] Purz S Mauz-Korholz C Korholz D Hasenclever D Krausse A Sorge I. [18F]Fluorodeoxyglucose positron emission tomography for detection of bone marrow involvement in children and adolescents with hodgkin's lymphoma. J Clin Oncol (2011) 29:3523–8. doi: 10.1200/JCO.2010.32.4996 21825262

[17] Zapata CP Cuglievan B Zapata CM Olavarrieta R Raskin S Desai K. PET/CT versus bone marrow biopsy in the initial evaluation of bone marrow infiltration in various pediatric malignancies. Pediatr Blood Cancer (2018) 65 (2). doi: 10.1002/pbc.26814 28901637

[18] Shah S Purandare N Kembhavi S Puranik A Agrawal A Bedmutha A. FDG PETCT for assessing marrow involvement at staging pediatric nonhematological round cell malignancies. Nucl Med Commun (2022) 43(1):56–63. doi: 10.1097/MNM.0000000000001491 34618718

[19] Pfluger T Piccardo A. Neuroblastoma: MIBG imaging and new tracers. Semin Nucl Med (2017) 47:143–57. doi: 10.1053/j.semnuclmed.2016.10.007 28237003

[20] Puccini B Nassi L Minoia C Volpetti S Ciancia R Riccomagno PC. Role of bone marrow biopsy in staging of patients with classical hodgkin's lymphoma undergoing positron emission tomography/computed tomography. Ann Hematol (2017) 96:1147–53. doi: 10.1007/s00277-017-2996-8 28451805

[21] Agool A Glaudemans AW Boersma HH Dierckx RA Vellenga E Slart RH. Radionuclide imaging of bone marrow disorders. Eur J Nucl Med Mol Imaging (2011) 38:166–78. doi: 10.1007/s00259-010-1531-0 PMC3005118 20625724

[22] Fan C Hernandez-Pampaloni M Houseni M Chamroonrat W Basu S Kumar R. Age-related changes in the metabolic activity and distribution of the red marrow as demonstrated by 2-deoxy-2-[F-18]fluoro-D-glucose-positron emission tomography. Mol Imaging Biol (2007) 9:300–7. doi: 10.1007/s11307-007-0100-9 17574502

[23] Liu J Li C Yang X Lu X Zhang M Qian L. The diagnostic value of (18)F-FDG PET/CT bone marrow uptake pattern in detecting bone marrow involvement in pediatric neuroblastoma patients. Contrast Media Mol Imaging (2022) 2022:7556315. doi: 10.1155/2022/7556315 35082556 PMC8758298

